# Late disruption of central visual field disrupts peripheral perception of form and color

**DOI:** 10.1371/journal.pone.0219725

**Published:** 2020-01-30

**Authors:** Kimberly B. Weldon, Alexandra Woolgar, Anina N. Rich, Mark A. Williams

**Affiliations:** 1 Department of Psychiatry and Behavioral Sciences, University of Minnesota, Minneapolis, MN, United States of America; 2 Perception in Action Research Centre (PARC), Department of Cognitive Science, Faculty of Human Sciences, Macquarie University, Sydney, NSW, Australia; 3 ARC Centre of Excellence in Cognition and its Disorders, Macquarie University, Sydney, NSW, Australia; 4 Medical Research Council (UK), Cognition and Brain Sciences Unit, University of Cambridge, Cambridge, England, United Kingdom; University of New England, AUSTRALIA

## Abstract

Evidence from neuroimaging and brain stimulation studies suggest that visual information about objects in the periphery is fed back to foveal retinotopic cortex in a separate representation that is essential for peripheral perception. The characteristics of this phenomenon have important theoretical implications for the role fovea-specific feedback might play in perception. In this work, we employed a recently developed behavioral paradigm to explore whether late disruption to central visual space impaired perception of color. In the first experiment, participants performed a shape discrimination task on colored novel objects in the periphery while fixating centrally. Consistent with the results from previous work, a visual distractor presented at fixation ~100ms after presentation of the peripheral stimuli impaired sensitivity to differences in peripheral shapes more than a visual distractor presented at other stimulus onset asynchronies. In a second experiment, participants performed a color discrimination task on the same colored objects. In a third experiment, we further tested for this foveal distractor effect with stimuli restricted to a low-level feature by using homogenous color patches. These two latter experiments resulted in a similar pattern of behavior: a central distractor presented at the critical stimulus onset asynchrony impaired sensitivity to peripheral color differences, but, importantly, the magnitude of the effect was stronger when peripheral objects contained complex shape information. These results show a behavioral effect consistent with disrupting feedback to the fovea, in line with the foveal feedback suggested by previous neuroimaging studies.

## Introduction

Visual object recognition is traditionally thought to conform to a bottom-up, feedforward model of processing that begins with the extraction of low-level object information in early visual areas [1,2]. From there, visual information proceeds along a hierarchy of cortical regions representing increasingly complex information. In addition, feedback connections from higher to lower visual areas also have an important role in visual perception, such that feedback modulates or attunes feedforward information [3–5]. Williams et al. [6] used multi-voxel pattern analysis of fMRI data to demonstrate that information about the category of novel objects [7] presented in the observer’s periphery during an object-discrimination task could be decoded in cortical regions that corresponded to central, foveal visual space, an area far removed from the stimulus input, in addition to expected object-selective regions (e.g. lateral occipital complex, LOC). The authors reasoned that object information at the foveal confluence was not a reflection of responses in object-selective regions because object information could be decoded from LOC but not the foveal region of interest during a color-discrimination task on the same stimuli. In other words, object information in foveal cortex was task-dependent while object information in object-selective regions was not. The authors attributed their findings to a feedback process, as the fovea remained unstimulated throughout the experiment. The results from Williams et al. [6] suggested a type of feedback mechanism that is capable of constructing a new and separate representation of peripheral object information, possibly reflecting a prediction related to a subsequent saccade to peripheral targets [8,9]. Critically, stronger representation of peripheral object category in foveal retinotopic cortex correlated with better behavioral performance on the task, implying an important role for this representation in perception.

A follow-up transcranial magnetic stimulation (TMS) study by Chambers, Allen, Maizey, and Williams [10] showed that integrity of the foveal region at a timeframe consistent with feedback is essential for peripheral perception. In that study, observers performed a task similar to that in Williams et al. [6]. Observers fixated centrally while discriminating between novel objects that briefly appeared in the observer’s periphery. A TMS pulse applied to the occipital pole selectively impaired perceptual discrimination sensitivity of peripheral objects when applied ~350ms *after* stimulus onset compared to a TMS pulse applied at other points in the course of a trial. TMS applied at stimulus onset asynchronies (SOAs) from 150ms prior to stimulus onset to 250ms post-stimulus onset, as well as beyond 400ms post-stimulus onset, did not have the same disruptive effect on discrimination sensitivity. Taken together, these studies suggest a form of feedback that constructs a representation of objects removed from the associated visual input and, further, that this feedback is behaviorally relevant, although more neuroimaging studies are needed to confirm this interpretation.

To date, studies examining the foveal feedback phenomenon have largely employed a relatively difficult behavioral task where the participants discriminate between briefly-presented novel greyscale objects [11,12]. However, in Williams et al. [6], the authors included one experiment where participants performed a color discrimination task on colored objects presented in the periphery. In that experiment, unlike the shape discrimination task, the authors did not find information about object form at the fovea, raising the possibility that foveal feedback is related to the task at hand. The authors did not, however, test whether color information could be decoded in foveal retinotopic cortex. Therefore, it is unknown whether foveal feedback is limited to carrying general shape information of visual stimuli, or if it may function for any one object characteristic related to the task being performed.

We have previously reported a measure designed to test for a behavioral consequence predicted by the notion of foveal feedback [12]. In brief, participants perform a discrimination task on achromatic novel objects briefly presented (~100ms) in their periphery while fixating centrally. We found that an achromatic visual distractor presented at fixation impairs discrimination sensitivity when it appears 117ms after target onset, after the targets have disappeared from the display. This disruption in discrimination sensitivity at +117ms post-stimulus onset reliably occurs when a central distractor is presented to the observer at a time entirely disparate from the target presentation, and is more pronounced compared to distractor onsets at other stimulus onset asynchronies (SOAs), including SOAs later in a trial (e.g., more than 250ms). In a previous paper, we termed this temporally-specific disruption of peripheral discrimination sensitivity the “foveal distractor effect” [12]. We also demonstrated the spatial specificity of this effect: discrimination sensitivity was not similarly impaired when a visual distractor was presented in the periphery at the critical SOA. This paradigm provided behavioural evidence consistent with the neuroimaging-based proposal of foveal feedback from the periphery. We therefore consider it an efficient method for investigating how feedback influences peripheral perception see also [11,13].

In the present set of experiments, we used the paradigm described in [12] to test whether the foveal distractor effect is specific to perceptual discrimination between object shapes, or if it also occurs during tasks requiring discrimination of another object characteristic, in this case, color. Color is a useful characteristic to use with these stimuli as its manipulation does not interfere with the fine spatial details of the novel objects. Although it is unknown whether color information about peripheral objects can be decoded from foveal retinotopic cortex during a color discrimination task [6], in light of electrophysiological research in monkeys suggesting cortical layers receiving feedback connections from higher visual areas may be selective for chromatic information [14], it seems plausible. This idea has been supported behaviorally: Zhaoping [15] used dichoptic stimuli to infer selectively stronger feedback to central vision rather than peripheral vision during color and tilt discrimination tasks. If feedback to foveal retinotopic cortex contains behaviorally-relevant information about peripheral objects that is not specific to form, then disruption to central visual space should disrupt discrimination sensitivity of objects in the viewer’s periphery when they perform both shape and color-discrimination tasks. Research on the cortical processing of color suggests that the neural computations related to form and color are strongly linked in early visual areas (for a review, see [16]). Early coupling of chromatic signals with other visual object characteristics such as orientation [17–19] and figure-ground segregation [20] have been well documented. Multi-voxel pattern analysis of fMRI data shows that object representation in early visual cortex does include information about the conjunction of color and object shape information [21]. We would therefore predict that, if our behavioral effect is not specific to complex form, we should also be able to evoke it with simple color patches.

In Experiment 1, we replicate the foveal distractor effect using colored novel objects (as opposed to achromatic objects as in [12]) in a task where participants discriminate between the objects’ shapes, while ignoring their colors. A central distractor presented 117ms after the onset of the targets impaired discrimination sensitivity of object shape in the periphery compared to distractors presented at SOAs very early or later in the trial. In Experiment 2, we used the same stimuli as in Experiment 1 but altered the task: participants were required to discriminate between the target colors while ignoring their shapes. To pre-empt our results, a visual distractor presented at fixation impaired peripheral discrimination sensitivity of color in the periphery, again only at the critical SOA. Finally, because in Experiment 2 the foveal distractor effect could occur due to disruption of bound color information to complex object form, in Experiment 3 we removed the complex shape information from the targets and had participants discriminate between circular colored patches. Overall, our results replicate the temporal specificity of the foveal distractor effect and show it is not unique to complex forms, but that the strength of the disruption is flexible and task-dependent.

## Experiment 1: Discriminating Form

### Materials and methods

#### Participants

Twenty participants, screened for normal or corrected-to-normal visual acuity as well as normal color vision using Ishihara color plates, were recruited for Experiment 1. One participant’s data were not used in the analysis due to chance-level performance, leaving the datasets of 19 participants (15 female, 4 male; mean age = 23.3 ± 4.55 years) for analysis. Participants received either course credit or $15 for their participation and gave informed consent. All experiments in this study were approved by the Macquarie University Human Research Ethics Committee.

#### Stimuli and apparatus

Sixteen stimuli were selected from a set of 1296 pre-generated “smoothie” stimuli [7]. These 16 exemplars were selected to represent the most extreme variations in the larger set. Using Matlab (Mathworks), each of the 16 exemplars was covered with a colored, transparent mask created in CIE L*c*h color space. Every colored mask had a luminance value of 85 and a chroma value of 38. The colored masks varied in hue angle from 0° (red) to 200° (blue) in steps of five degrees, resulting in a full stimulus set of 656 objects. We used a large range of colors to mimic the variability in the shapes of the exemplars. A further smoothie stimulus, which was not one of the 16 main exemplars, was selected for use as a visual distractor. This distractor was covered with a colored mask that had a hue value of 63°, which was not one of the possible target colors. In this way, it was possible for the distractor object to vary in degree of similarity, to the color and/or shape of either target while never being identical to either characteristic. Each stimulus subtended ~1.5° of visual angle.

Experimental sessions took place in a dimly-lit, windowless laboratory at Macquarie University, Sydney. Stimuli were presented on an sRGB-calibrated 27in Samsung SyncMaster AS950 monitor at a resolution of 1920x1080 pixels and a refresh rate of 120Hz. We tracked fixation of the right eye with an Eyelink 1000 remote eye-tracker at 500Hz. The camera and infrared illuminator were mounted in front of the participant below the desktop display so that the screen was not obscured.

#### Training procedure

**Fig 1:**
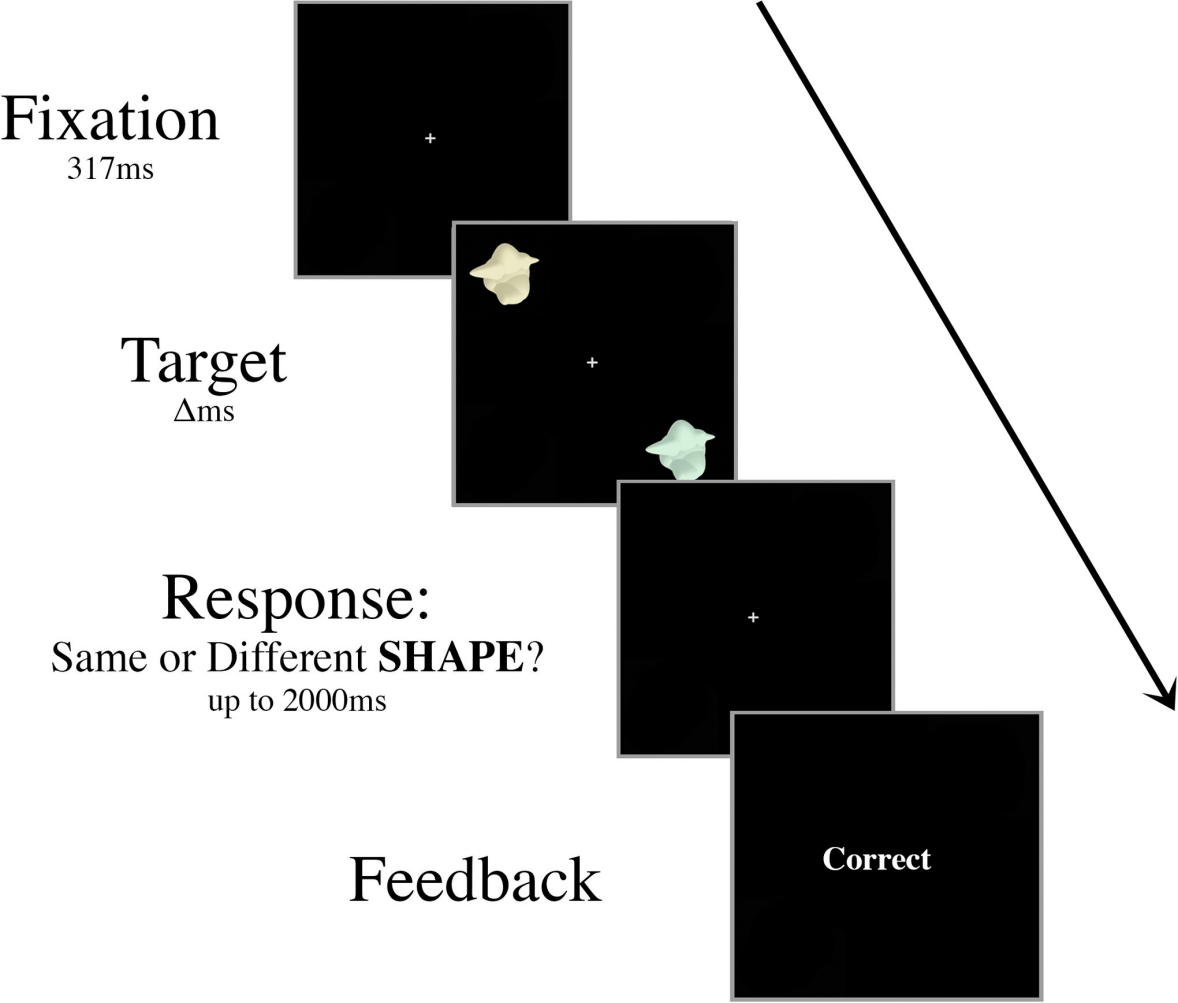
Schematic of an example “same” trial in the Experiment 1 training task. Targets were presented for decreasing durations (Δ: 417ms, 267ms, then 117ms) during training. Participants were instructed to ignore the color of the targets and judge only if the shapes of the targets are identical. In this example, the two targets are different colors but the same shape, requiring a “same” response. Training continued until the participant was able to perform above 70% accuracy with a 117ms presentation time across a single block of ten trials.

Prior to the experiment, participants were trained on a basic discrimination task (with no central distractor). First, a white fixation cross was displayed for 315ms. Then, two colored target objects were displayed for 417ms in the upper left and lower right quadrants of the screen. The targets were presented in these same locations throughout the training tasks and the experiments (Fig 1). Participants were instructed to maintain fixation on the central cross throughout each trial and determine if the two targets in the array were different shapes or if they were identical in shape as quickly and accurately as possible, while ignoring the color of the targets. Participants had 2000ms to respond with their right index finger or middle finger on the keyboard to indicate a “same” or “different” judgment, respectively. Following each response, participants were given onscreen accuracy feedback. After a 2000ms interstimulus interval, the next trial commenced automatically.

In half of the trials, these target stimuli were different shapes, chosen at random from the larger set of 16, and in the other half they were identical shapes. The targets, regardless of whether they were the same or different shapes, always differed in color by a hue angle of 60°. This ensured that participants were not able to use color as a cue in shape discrimination. The degree of color difference (60°) was selected based on pilot data to set participants’ performance on a shape discrimination task as similar to their performance in a color discrimination task using the same stimuli (see Experiment 2). Trials where the participant’s eye gaze drifted more than 2° from the center of the display were coded as incorrect during training.

Trials were presented in blocks of ten. Once participants could perform the discrimination task with >70% accuracy across a single block with a target display duration of 417ms, the presentation time of the targets decreased to 267ms. Participants repeated the training procedure until they were able to perform the task with >70% accuracy in a block. Then, the presentation time of the targets further decreased to 117ms, which reflected the timing conditions in the experiment. Training continued until participants were able to make at least 70% correct discriminations when the target array was displayed for 117ms, while maintaining fixation throughout the block. In general, participants were able to complete the training within 20 minutes.

#### Experimental procedure

**Fig 2:**
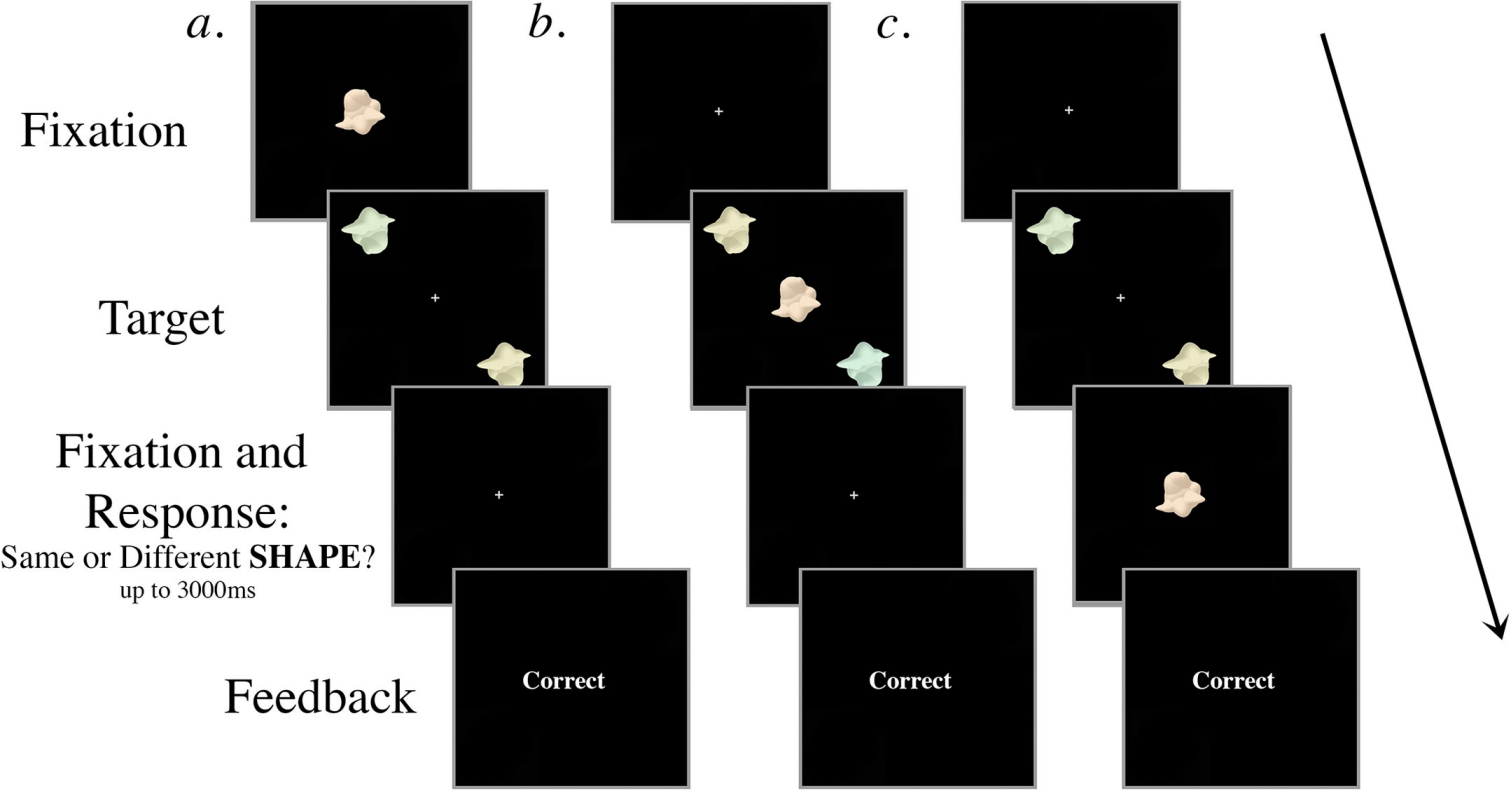
Schematic of 3 example trials in Experiment 1 with a colored distractor. Participants judged whether the peripheral targets were the same shape, ignoring their colors, which were always different. The targets and the distractor were displayed for 117ms regardless of SOA. The central distractor appeared either (a) 267ms or 117ms prior to target onset, (b) simultaneously with target onset, or (c) 117ms or 267ms after target onset. In the examples shown, the targets are different colors but identical shapes and the correct response is “same”. (In Experiment 2, participants judged whether the targets were the same color, ignoring their shapes; for these displays in Experiment 2 the correct response would be “different”).

The procedure for Experiment 1 was similar to the training procedure with two major changes: there was a fixed target presentation duration of 117ms and a distractor object appeared at fixation once during each trial (Fig 2). At the beginning of each trial, a white central cross was displayed for 567ms. In each target display, two colored targets were displayed for 117ms in opposite diagonal locations (upper left and lower right-hand quadrants of the screen), each at 6.5° eccentricity. The targets were identical shapes in half the trials and different shapes in the other half, randomly selected from the set of 16 exemplars. As in the training trials the colors of the two targets, in both “same” and “different” trials, always differed by a hue angle of 60°.

At one point in each trial, a distractor object appeared at fixation for 117ms. There were ten trial conditions that dictated the timing and the type of the distractor presented. First, the onset of the distractor object occurred at one of five possible SOAs: 267ms prior to target onset (-267ms), 117ms prior to target onset (-117ms), simultaneously with target onset (0ms), 117ms after target onset (+117ms), or 267ms after target onset (+267ms). Second, the distractor was either greyscale or colored with a hue angle of 63°. Since the targets varied in hue angle from 0° to 200° in steps of five degrees (0°, 5°, 10°, etc.), the hue angle of the colored distractor differed from that of the targets by between 2° and 137°, depending on the color of the targets. There were 80 trials for each of the ten conditions (40 “same”, 40 “different”) for a total of 800 trials in a session. All of the trial types were randomly intermingled, fully crossed, and blocked so that participants would have a chance to rest every 100 trials.

Participants were given 3s to respond after the completion of the trial before the next trial automatically commenced. As in the training task, participants used their right index finger to indicate a “same” judgment or their right middle finger to indicate a “different” judgment. Following each response, participants were given onscreen accuracy feedback.

As in training, participants were instructed to maintain fixation on the central cross throughout each trial and respond as quickly and accurately as possible. The eye-tracker was unavailable for six participants. However, given the short duration of the target display as well as the disparate peripheral target locations, any eye-movements towards the peripheral stimuli are likely to have impaired behavioral performance on the task, as only a single target would be able to be fixated (if that) during the display, which would make the second target further from fixation, making it more difficult to compare the two stimuli. In the cases of eye-tracked participants, we had to discard only 0.08% of completed trials from analysis due to eye-movements. Participants were able to complete the experimental task in ~45 minutes.

We did not include a non-distractor condition in the main experiment because the training task was effectively the discrimination task without a distractor. Additionally, a non-distractor condition differs from the experimental distractor-present condition. Thus, a ‘no-distractor’ condition would not be a good baseline as performance could be better due simply to practice or the other changes. Instead, we used performance in the -267ms SOA condition as a baseline for comparison as it is matched the experimental conditions in all key aspects with the only difference being the onset time of the distractor.

### Results

**Fig 3:**
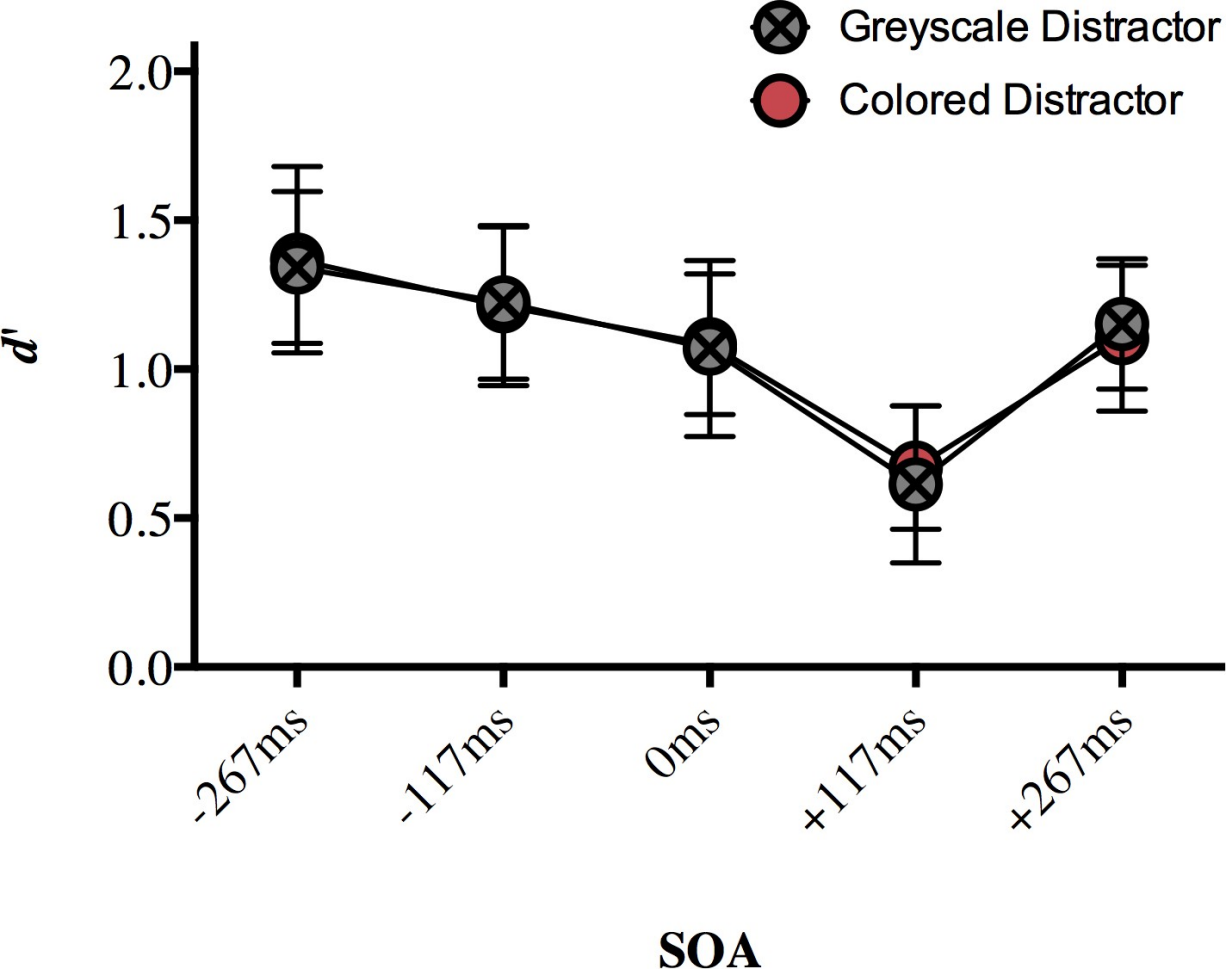
The effect of a central distractor on peripheral color discrimination (mean d’) in Experiment 1. A distractor appearing 117ms after target onset disrupted target discrimination sensitivity more than distractors appearing at every other SOA. Error bars represent 95% confidence intervals. Significant differences are discussed in text.

Our dependent variable was *d’* as a measure of discrimination sensitivity for comparing the targets. The hit rate was defined as the proportion of correct “same” responses on “same” trials, and the false alarm rate was defined as the proportion of “same” responses on “different” trials (S1 Table). We ran a two-way repeated measures ANOVA on *d’* with the factors of SOA (-267ms, -117ms, 0ms, +117ms, +267ms) and distractor type (grey, colored). We applied a Greenhouse-Geisser correction to the main effect of SOA in order to correct for violated sphericity found using Mauchly's Test of Sphericity (χ^2^(9) = 21.215, *p* = 0.012). There was a significant main effect of SOA (*F*(2.75, 49.56) = 20.258, *p* < 0.001, η_p_^2^ = 0.530), no main effect of distractor type (*F*(1, 18) = 0.042, *p* = 0.841, η_p_^2^ = 0.002), and no interaction (*F*(4, 72) < 1, *p* = 0.970, η_p_^2^ = 0.007; Fig 3). This result demonstrates that discrimination sensitivity varies with SOA, and whether the distractor object was colored or greyscale does not seem to affect participants’ ability to discriminate between peripheral colored objects.

A Bonferroni correction for multiple comparisons (α = 0.05/10 = 0.005) was applied to post hoc analyses following up the main effect of SOA (data collapsed over distractor type). For our key SOA of +117ms, discrimination sensitivity (*d*’) was impaired compared to our relative baseline -267ms (*p* < 0.001), as well as compared to -117ms (*p* < 0.001), 0ms SOA (*p* < 0.001) and +267ms SOA (*p* < 0.001; Fig 3). The only other significant difference was that discrimination sensitivity was significantly lower at 0ms SOA than -267ms SOA (*p* < 0.001). No other comparisons approached significance after correction (*p* > 0.005; S2 Table). Taken together, these results show that a central distractor appearing 117ms after target onset disrupted participants’ ability to discriminate between the peripheral targets more than a distractor appearing at other SOAs. This is an important replication of the foveal distractor effect [12] with stimuli that have different features.

## Experiment 2: Discriminating color

Most studies investigating the temporally-specific disruption of peripheral discrimination sensitivity have used a task requiring discrimination of fine spatial details ([8,10,12] but see [11]). The aim of Experiment 2 was to determine whether this foveal distractor effect would occur when participants attend to and perform a discrimination task on an object characteristic other than shape, in this case, color. Color is an object characteristic that is easily manipulated while avoiding changes to spatial details of the visual stimuli. We used the stimuli from Experiment 1 in order to minimize differences between the two experiments.

### Materials and methods

#### Participants

A naïve group of 20 participants was recruited for Experiment 2. One participant’s dataset was discarded due to chance-level performance, leaving 19 full datasets for analysis (15 female, 4 male; mean age = 21.34 ± 5.06 years). Participants reported normal or corrected-to-normal visual acuity, were screened for normal color vision using Ishihara color plates, and gave informed consent. Each received course credit or $15 for their participation.

#### Procedure

The stimuli and apparatus were the same as in Experiment 1. Prior to taking part in the experiment, participants were trained on a basic discrimination task similar to the training for Experiment 1, except that in Experiment 2, participants discriminated between the colors rather than the shapes of the objects. The shapes of the target objects in Experiment 2 were always different, randomly chosen from the set of 16 exemplars. This ensured participants could not use shape as a cue in the color discrimination decision. Participants were instructed to ignore the shapes of the targets and make a judgement on whether the colors of the targets were identical or different. In each trial, one color was chosen pseudorandomly between the hue angles of 0° and 200°. As described in Experiment 1, this number was in a multiple of 5 (5°, 10°, 15°, etc.) to increase the variability of target colors. In “same” trials, the objects’ colors were identical. In “different” trials, the colors always differed by a hue angle of 60°. The degree of difference was determined based on pilot data such that participants would be able to discriminate between the two colors with a similar accuracy as when doing the shape task described in Experiment 1, and the range of colors was chosen to complement the variability in the shapes of the exemplars. The parameters of the training task were the same as in Experiment 1 (see Fig 1). Participants were trained until they were able to make at least 70% correct discriminations when the target array was displayed for 117ms, while maintaining fixation throughout the block.

Experiment 2 was carried out in a similar way to Experiment 1 (see Fig 2), with the exception that participants were asked to judge whether the two colored objects were the same *color* while ignoring their shapes. As in Experiment 1, the distractor was either grayscale or covered with a colored mask that had a hue value of 63°. The hue angle of the colored distractor differed from that of the targets by between 2° and 137°, depending on the color of the targets.

The eye-tracker was unavailable for seven of the participants in Experiment 2. In the cases of eye-tracked participants, we discarded 0.08% of completed trials from analysis.

### Results

**Fig 4:**
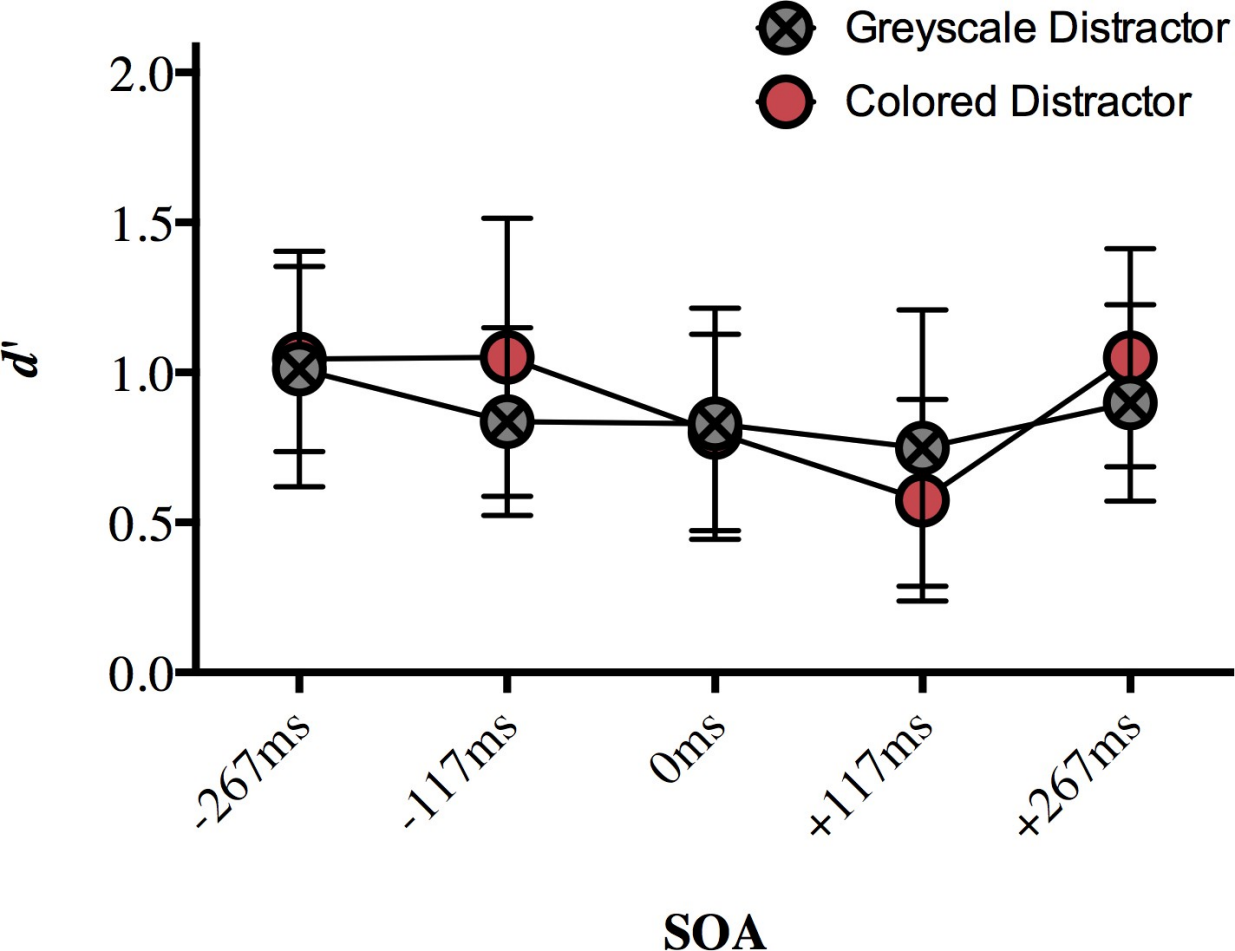
The effect of a central distractor on peripheral color discrimination (mean d’) in Experiment 2. Error bars represent 95% confidence intervals. Significant differences are discussed in text.

Our dependent variable was again *d’* for target discrimination sensitivity. The hit and false alarm rates (S3 Table) were defined as in Experiment 1. We ran a two-way repeated measures ANOVA on *d’* with the factors of SOA (-267ms, -117ms, 0ms, +117ms, +267ms) and distractor type (grey, colored). There was a significant main effect of SOA (*F*(4, 72) = 7.328, *p* < 0.001, η_p_^2^ = 0.289), no main effect of distractor type (*F*(1, 18) = 1.045, *p* = 0.32, η_p_^2^ = 0.55), and no interaction (*F*(4, 72) = 1.918, *p* = 0.117, η_p_^2^ = 0.096; Fig 4). This result suggests that target discrimination sensitivity on the color task varied with distractor SOA, and whether the distractor object was colored or grey had little effect on performance.

A Bonferroni correction for multiple comparisons (α = 0.05/10 = 0.005) was applied to post hoc analyses following up the main effect of SOA (data collapsed over distractor type). Target discrimination sensitivity was significantly impaired for +117ms SOA compared to that at -267ms SOA (*p* = 0.001), -117ms SOA (*p* = 0.003), and +267ms SOA (*p* < 0.001). No other comparisons survived correction (*p* > 0.021; S4 Table). Although the pattern is less clear for this experiment, the significant results are similar to the pattern of results from Experiment 1, where a central distractor appearing 117ms after target onset disrupted participants’ ability to discriminate between the peripheral targets more than a target appearing at other non-simultaneous SOAs. The main discrepancy is the lack of a difference between 0ms SOA and +117ms SOA, which does not come out in this experiment; being a null effect, we will not interpret this further.

## Experiment 3: Color discrimination with simple shapes

In Experiment 2, participants discriminated between the colors of novel objects. A distractor object in central vision at 117ms post-stimulus onset impaired target discrimination sensitivity relative to most of the other SOAs (except 0ms). This result suggests that feedback to foveal retinotopic cortex carries task-relevant information (in this case, color). However, the targets in Experiment 2, being novel objects, still contained complex shape information. It is therefore possible that it is not the relevance of color that drove the result, *per se*, but instead the link between the shape and color [16,17,21]. The aim of Experiment 3 was to see whether the effect at the critical SOA remained when the stimuli were restricted to a single low-level feature (color) and participants therefore did not have to ignore any aspect of the targets.

### Materials and methods

#### Participants

A naïve group of 20 participants was recruited for Experiment 3 (11 female, 9 male; mean age = 21.5 ± 3.99 years). Participants received either course credit or $15 for their participation. All participants were screened for normal color vision, and normal or corrected-to-normal visual acuity and gave informed consent.

#### Stimuli and apparatus

**Fig 5:**
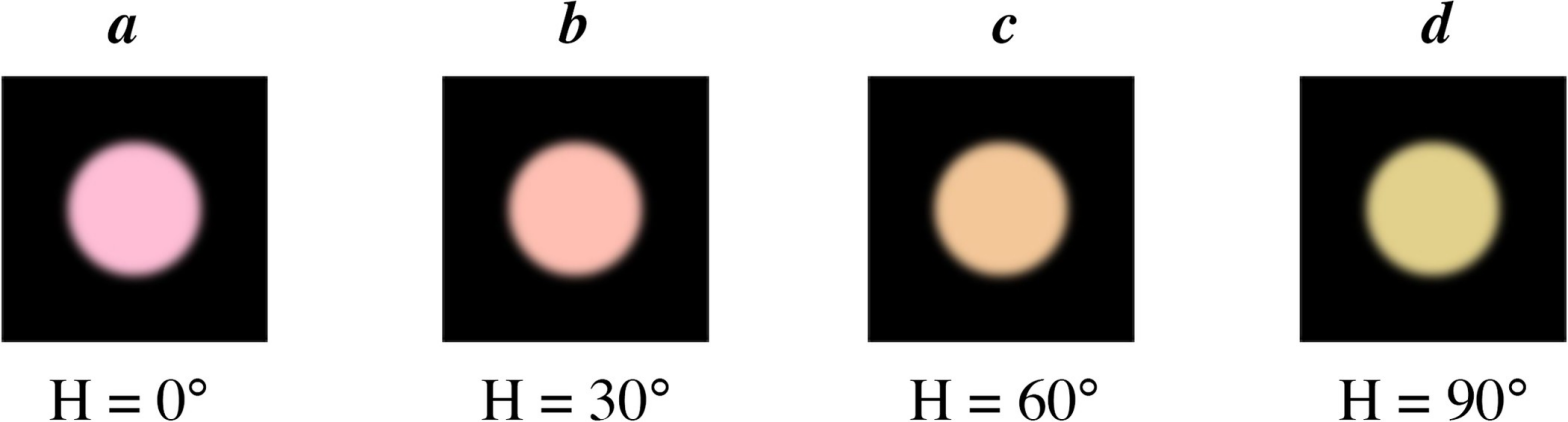
Examples of stimuli used as targets in Experiment 3. Exemplars differed by 30°, so that, for example, (a) and (b), (b) and (c), or (c) and (d) could be used as pairs. Only hue angle varied; luminance and saturation remained constant.

All aspects of the apparatus were the same as Experiments 1 and 2. The stimuli were a set of color patches, presented on a black background, using the same luminance (85), chroma (38), and hue values (0°-200° in steps of five) from Experiments 1 and 2. Using Matlab, the original circles (*r* = 125 pixels) were filtered with a rotationally symmetric Gaussian low-pass filter of size 100 x 100 with a standard deviation of 10 (Fig 5). In the experiment, the targets were sized to subtend ~1.5° visual angle as in the previous experiments. For this experiment, the targets in “different” trials differed in color by a hue angle of 30°, rather than 60° in Experiments 1 and 2. The degree of color difference was chosen based on pilot data, such that participants’ performance on the color discrimination task with these simpler stimuli (that do not require them to ignore an irrelevant feature) would be similar to performance in the shape discrimination task described in Experiment 1. The distractor was either a greyscale color patch or a color patch with a hue angle of 63°. The hue angle of the colored distractor differed from that of the targets by between 2° and 137°, depending on the color of the targets.

#### Procedure

In Experiment 3, participants were asked to judge whether the two target circles were the same or different colors. This meant that unlike in the previous experiments, they were no longer required to ignore any feature of the targets. Otherwise, the training and experimental procedures were the same as in Experiment 2. Three participants were not eye-tracked due to technical problems with the eye-tracker. For the other participants, we discarded 0.06% of the eye-tracked trials from the analysis due to fixation failures.

### Results

**Fig 6:**
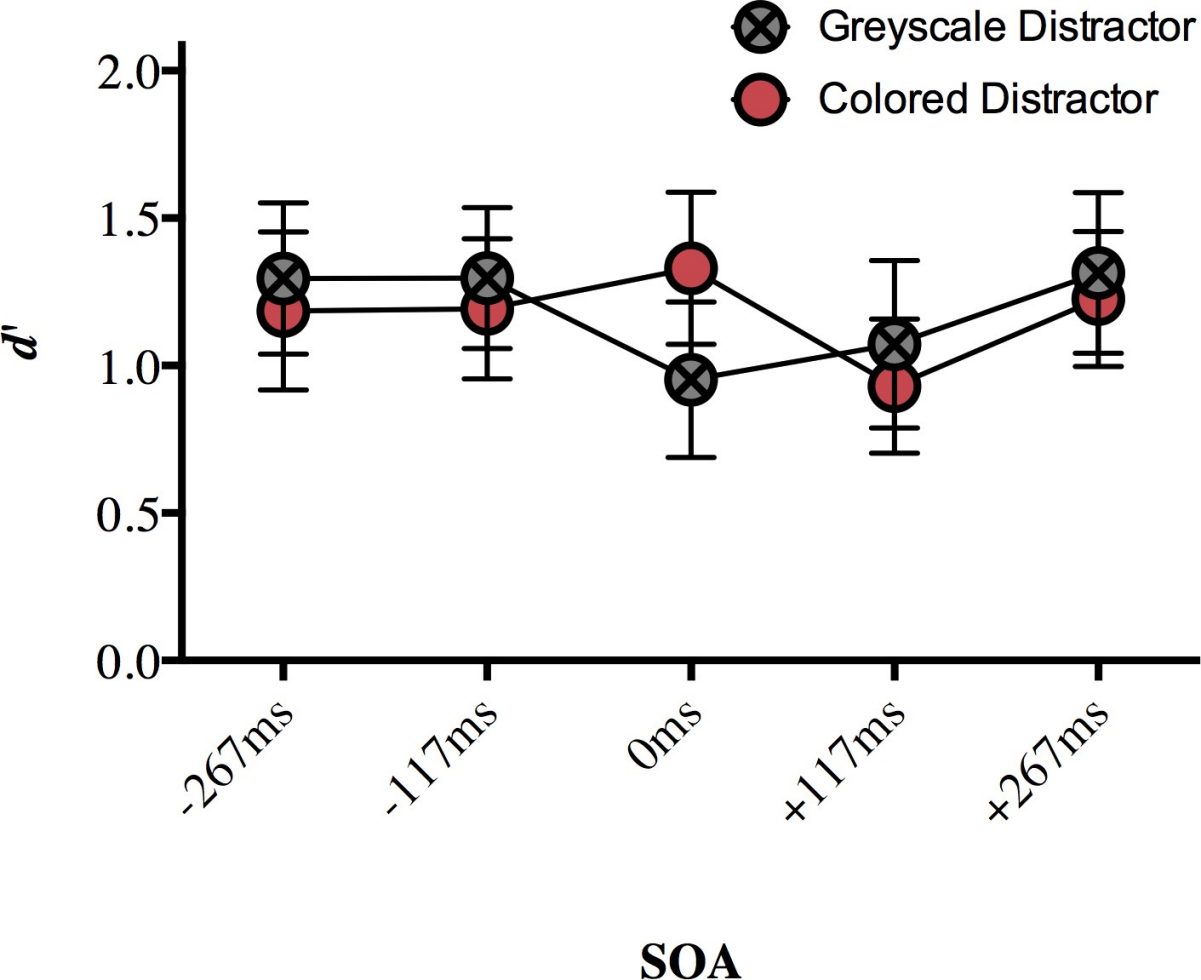
The effect of a central distractor on peripheral color discrimination (mean d’) in Experiment 3. Error bars represent 95% confidence intervals. Significant differences are discussed in text.

The dependent variable was again *d’* for target discrimination sensitivity. The hit and false alarm rates (S5 Table) were defined as in Experiments 1 and 2. A two-way repeated measures ANOVA on *d’* with the factors of SOA (-267ms, -117ms, 0ms, +117ms, +267ms) and distractor type (greyscale, colored) showed a significant main effect of SOA (*F*(4, 76) = 4.373, *p* = 0.003, η_p_^2^ = 0.187), no effect of distractor type (*F*(1, 19) = 0.117, *p* = 0.736, η_p_^2^ = 0.006), and a significant interaction (*F*(4, 76) = 4.075, *p* = 0.005, η_p_^2^ = 0.177; Fig 6).

We followed up the interaction with a repeated measures ANOVA on the distractor type conditions separately (Fig 6). There was a main effect of SOA for both the colored distractor (*F*(4, 72) = 9.659, *p* < 0.001, η_p_^2^ = 0.349) and greyscale distractor (*F*(4, 72) = 13.026, *p* < 0.001, η_p_^2^ = 0.42; Fig 6) conditions. A Bonferroni correction for multiple comparisons (α = 0.05/20 = 0.0025) was applied to the post hoc analyses. For the colored distractor condition, target discrimination sensitivity was impaired with the distractor was presented at +117ms SOA compared to a distractor presented at 0ms SOA (*p* = 0.002; Fig 6). The difference in mean *d’* values for +117ms and -267ms (*p* = 0.058), -117ms (*p* = 0.013), and +267ms (*p* = 0.003) did not reach significance after correction but suggest a pattern of results similar to that demonstrated in Experiments 1 and 2 (S6 Table). No other comparisons approached significance (*p* > 0.05).

For the greyscale condition, discrimination sensitivity was significantly impaired for 0ms SOA compared to -267ms SOA (*p* = 0.001). Mean *d’* values for +117ms SOA were numerically lower than mean *d’* values at -267ms SOA (*p* = 0.019), -117ms SOA (*p* = 0.007), and +267ms SOA (*p* = 0.019) but these differences did not reach significance after correction (Fig 6).

We also followed up the interaction of SOA and distractor type by examining the effect of distractor type at each SOA separately. Post-hoc analyses of the interaction using a Bonferroni correction (α = 0.05/10 = 0.005) showed that a greyscale distractor impaired target discrimination sensitivity relative to a colored distractor (*p* = 0.004; Fig 6). At all other SOAs, there was no significant effect of the color of the distractor type (*p >* 0.05; S7 Table).

## Discussion

The aim of this study was to test whether the foveal distractor effect is limited to form-related information or extends to other visual features. In Experiment 1, we replicated the effect first demonstrated with achromatic stimuli in Weldon et al. [12] with colored novel objects. When participants were asked to discriminate the shape of two peripheral target objects while ignoring their color, a distractor presented at fixation at +117ms SOA impaired perceptual discrimination more than a distractor presented at SOAs very early or late in the trial. At the critical SOA (117ms), the targets are no longer present onscreen and the distractor appears in an entirely different location from that of the target array. We also demonstrated the foveal distractor effect in Experiment 2, where participants were required to ignore the target shape (which were always different), and discriminate target color on the same set of stimuli. Although the results were not as clear as in Experiment 1, we do see a demonstration of the foveal distractor effect during a color discrimination task.

In Experiment 3, we minimized shape information in the stimuli by presenting homogenous color patches as targets. In the colored distractor condition, we found target discrimination sensitivity was impaired with a delayed distractor presentation (+117ms SOA) compared to a distractor presented at 0ms SOA, replicating the basic effect. Unlike in Experiment 1 and 2, however, Experiment 3 had an interaction of distractor type and SOA. A greyscale distractor was more disruptive to discrimination sensitivity than a colored distractor when it was presented simultaneously with the targets (and only then). This finding differs somewhat from previous work where participants performed a discrimination task on achromatic versions of the stimuli used in this paper. A central, inconsistent distractor (an angular, “cubie” version of the target stimuli, see [7]) did not interfere with peripheral discrimination sensitivity more than a distractor that was consistent with the targets (an object from the same shape category) [12]. Here, although the colored distractor was always a different hue than the targets, it could be considered more “consistent” with colored targets than a greyscale distractor. In that light, the finding in Experiment 3 is consistent with research suggesting that central distractors that are unrelated to targets cause more interference in visual search tasks than when the central distractor is identical to the target [22].

It is unclear why distractor type differentially influenced behavioral performance in the “simple” color discrimination task at 0ms SOA and not in Experiments 1 and 2. Any distractor appearing simultaneously with the distractors could reasonably be expected to interfere with peripheral perception simply because there is more information present in the visual field (see Experiment 1; [12]. One account for the finding could be from computational differences related to the chromaticity of the distractor [23], the requirement to discriminate between a feature with low spatial frequency rather than complex spatial information [11], or some combination of these possibilities. Introducing more structure into the simple stimuli by using gratings instead of homogenous patches would shed light on how the complexity of the stimuli influences the disruptive potential of a distractor. It also would be interesting to test how the disruptive effect of the distractor would change with a reduction in the distractor’s predictability (e.g. using a range of distractor hue values). The finding at 0ms SOA is unexpected, but not directly relevant to our main investigation regarding feedback at later SOAs.

The timecourse of the effect for this particular paradigm has been consistent across multiple experiments (see [12]) and with various masking paradigms used to examine visual feedback [24,25]. However, it is earlier than the timecourse reported in the TMS study targeting the foveal feedback phenomenon [10] and other behavioral paradigms designed to target the effect [11,13]. A TMS pulse has a fairly immediate effect on its targeted cortical sites, whereas a visual distractor must go through the usual feedforward processes of the visual system (~60ms-90ms; [26]). It is possible that the discrepancy between the critical timecourse demonstrated here (+117ms SOA) and the TMS experiment [10] is related to differences in the behavioral task. The presentation duration of the target stimuli in Chambers et al. [10] was titrated so that the task was equally difficult for all of the participants. Here, the duration of target presentation was maintained at 117ms throughout the experiment, resulting in variance in overall accuracy. It is plausible that such differences in difficulty could affect the exact timecourse of feedback: perceptually more difficult tasks may take longer to be fed back to the foveal confluence. For example, Fan et al. [11] showed that foveal interference occurred around 450ms SOA in a task involving both mental rotation and discrimination in the same trial. Taken together, these studies and ours suggest that the type of task or level of task difficulty has the potential to influence the timecourse of feedback signals, and thus, the time at which our foveal distractor effect would be evident.

An alternative possibility is that, in this paradigm, the distractor at +117ms SOA is targeting or impairing ongoing feedforward processes associated with discrimination of the targets rather than feedback signals. For instance, the distractor at the critical SOA might have impaired discrimination simply by capturing attention (based on feature-based attention effects [27]) or by masking object-selective neurons responding to the targets. Under this view, however, the simultaneous presentation of distractor with the targets has a higher likelihood of capturing disruptive effects beyond feedback (like feature-based attention). The increased decrement at +117ms compared to 0ms SOA in Experiments 1 and 3 is difficult to reconcile with a purely feedforward disruption by the distractor at the delayed SOA.

The paradigm used in the present experiments can be considered a variation of backwards masking paradigms. Backward masking occurs when the perception of a target stimulus is impaired or eliminated by the appearance of a second stimulus (the mask) after the presentation of the target. Under the model of masking put forth by Lamme and Roelfsema [28], the presentation of a mask following the target disrupts the feedback from high to low visual areas, thereby disrupting perception of the target. One criticism of using a feedback model to explain backward masking effects is that the temporal characteristics of backward masking paradigms have not been sufficiently tested [29]. In the studies presented here, the *offset* of the target array coincides with the *onset* of the distractor at the critical SOA of +117ms. Although there was no overlap between the display time of the target array and the display time of the distractor at 117ms SOA, we cannot be sure, based on the limited amount of literature using this paradigm, the extent to which the co-occurrence of offset of the target array with the onset of the distractor affected performance. Further experiments are required to comprehensively map out the timecourse of the effects reported here and elsewhere [11–13].

The spatial specificity of the foveal distractor effect has been tested previously [12]. In that study, the targets were also placed in the upper left and lower right quadrants of the visual field. A distractor placed in the periphery (upper right quadrant) did not impair discrimination sensitivity at the critical SOA compared to a distractor placed at the fovea. Although we did not attempt to replicate that finding in the present set of experiments, the experimental design is very similar and we would expect to find the same level of spatial specificity. When testing for spatial specificity of foveal feedback in an fMRI study, Williams et al. conducted an experiment where targets were placed in the upper left and upper right quadrants of the display. Using MVPA, that group reported that object category information could be decoded at the foveal confluence, and object information could not be decoded at the midpoint between targets. A visual distractor placed at the midpoint between targets in the same hemifield would be closer to the targets than peripheral distractors in Weldon et al. [12], and have more potential to impair ongoing object discrimination processes [30]. To fully evaluate the spatial specificity of the foveal distractor effect, future behavioral studies need to test the disruptive effect of a distractor placed at other peripheral locations, particularly when the targets are placed in the same hemifield. Such studies would also help link the behavioral paradigm employed here to neuroimaging investigations of foveal feedback.

Overall, the present experiments demonstrate that the foveal distractor effect is not specific to object shape information. If the behavioral effect described here reflects the foveal feedback phenomenon observed in neuroimaging data [6,10], these results predict that feedback to the foveal confluence is important for the peripheral discrimination of color as well as shape, especially when discriminating between complex colored shapes. The possibility that foveal feedback supports color discrimination in the periphery is consistent with behavioral findings inferring robust feedback to central vision in a color discrimination task using dichoptic multicolored gratings [15], which contained more spatial detail than the homogenous patches used in Experiment 3. In that vein, the more subtle findings from discrimination of homogenous color patches would suggest that the foveal feedback signal is flexible and primarily used in tasks involving discrimination between fine spatial detail compared to other tasks [11]. It could also be related to task difficulty; Experiments 1 and 2 required participants actively ignore one characteristic of the targets, while Experiment 3 did not. A next step would be to systematically interrogate how the level of complexity in a stimulus affects evoked foveal feedback.

Does decodable information at foveal retinotopic cortex vary with task type, stimulus complexity, or both? In Williams et al. [6], object information in foveal cortex was present only when participants performed the object discrimination task, but not when they performed a color discrimination task on the same stimuli. We need neuroimaging studies to determine whether color information is likewise present at foveal retinotopic cortex only during a behavioral task requiring color discrimination, as predicted by our behavioral effects. Such studies would be able to address the question of whether irrelevant information is fed back to foveal cortex (which is more difficult to measure behaviorally). That said, the evidence from the present set of experiments that perception of peripheral object form and color is affected by delayed disruption of central visual space, lends credence to the proposal that foveal cortex serves to store or compute task-relevant visual information for a range of perceptual tasks.

## Supporting information

S1 Fig Response times for Experiment 1. A two-way repeated measures ANOVA on Response Time with the factors of SOA (-267ms, -117ms, 0ms, +117ms, +267ms) and distractor type (greyscale, colored) showed a significant main effect of SOA (*F*(4, 72) = 7.718, *p* < 0.001), no effect of distractor type (*F*(1, 18) = 0.079, *p* = 0.782), and no interaction (*F*(4, 72) = 1.57, *p* = 0.193).
(PDF)

S2 Fig Response times for Experiment 2. A two-way repeated measures ANOVA on Response Time with the factors of SOA (-267ms, -117ms, 0ms, +117ms, +267ms) and distractor type (greyscale, colored) showed a significant main effect of SOA (*F*(4, 72) = 37.437, *p* < 0.001), no effect of distractor type (*F*(1, 18) = 3.253, *p* = 0.088), and no interaction (*F*(4, 72) = 0.839, *p* = 0.505).
(PDF)

S3 Fig Response times for Experiment 3. A two-way repeated measures ANOVA on Response Time with the factors of SOA (-267ms, -117ms, 0ms, +117ms, +267ms) and distractor type (greyscale, colored) showed a significant main effect of SOA (*F*(4, 72) = 36.272, *p* < 0.001), a significant main effect of distractor type (*F*(1, 18) = 5.23, *p* = 0.034), and no interaction (*F*(4, 72) = 1.133, *p* = 0.348).
(PDF)

S1 Table Hit and false alarm proportions in Experiment 1: Discriminating form. The Hit Rate was defined as the proportion of correct “same” responses on “same” trials, and the False Alarm Rate was defined as the proportion of “same” responses on “different” trials. Trials where eye-tracked participants failed to maintain fixation were not included in the final analysis.
(PDF)

S2 Table Experiment 1: Discriminating form analysis. Asterisks indicate significance after Bonferroni correction for multiple comparisons (α = 0.05/10 = 0.005).
(PDF)

S3 Table Hit and false alarm proportions in Experiment 2: Discriminating color. The Hit Rate was defined as the proportion of correct “same” responses on “same” trials, and the False Alarm Rate was defined as the proportion of “same” responses on “different” trials. Trials where eye-tracked participants failed to maintain fixation were not included in the final analysis.
(PDF)

S4 Table Experiment 2: Discriminating color analysis. Asterisks indicate significance after Bonferroni correction for multiple comparisons (α = 0.05/10 = 0.005).
(PDF)

S5 Table Hit and false alarm proportions in Experiment 3: Color discrimination with simple shapes. The Hit Rate was defined as the proportion of correct “same” responses on “same” trials, and the False Alarm Rate was defined as the proportion of “same” responses on “different” trials. Trials where eye-tracked participants failed to maintain fixation were not included in the final analysis.
(PDF)

S6 Table Experiment 3: Color discrimination with simple shapes data analysis A. Asterisks indicate significance after Bonferroni correction for multiple comparisons (α = 0.05/20 = 0.0025).
(PDF)

S7 Table Experiment 3: Color discrimination with simple shapes data analysis B. Asterisks indicate significance after Bonferroni correction for multiple comparisons (α = 0.05/10 = 0.005).
(PDF)
