# Supplementary figures and images for: Late disruption of central visual field disrupts peripheral perception of form and color
Source: PLoS One. 2020 Jan 30;15(1):e0219725. doi: 10.1371/journal.pone.0219725 (PMC6991998; doi:10.1371/journal.pone.0219725)

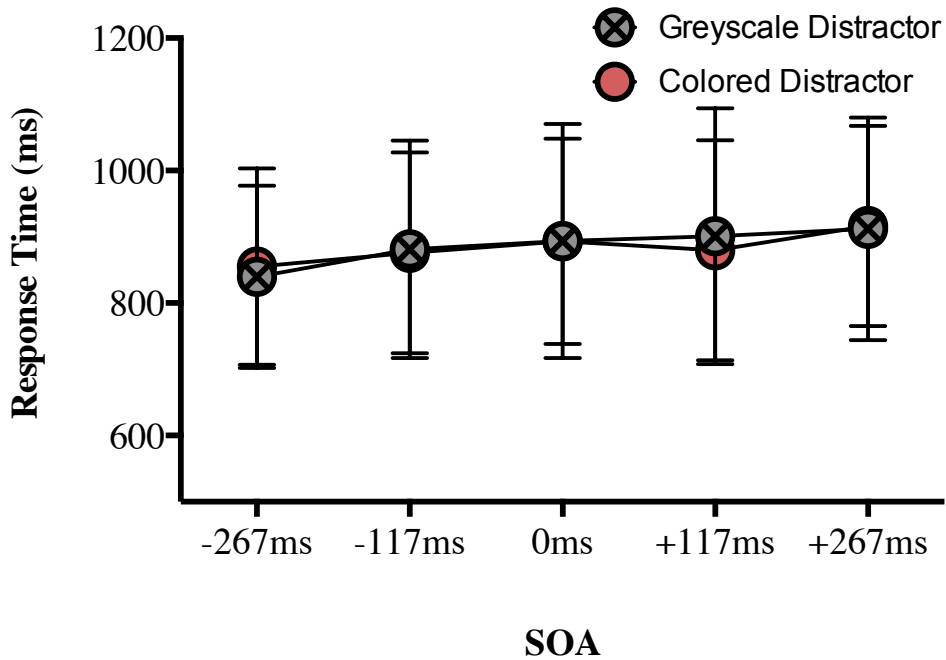

Supplement: S1 Fig — A two-way repeated measures ANOVA on Response Time with the factors of SOA (-267ms, -117ms, 0ms, +117ms, +267ms) and distractor type (greyscale, colored) showed a significant main effect of SOA (F(4, 72) = 7.718, p < 0.001), no effect of distractor type (F(1, 18) = 0.079, p = 0.782), and no interaction (F(4, 72) = 1.57, p = 0.193). (PDF) [file pone.0219725.s001.pdf]

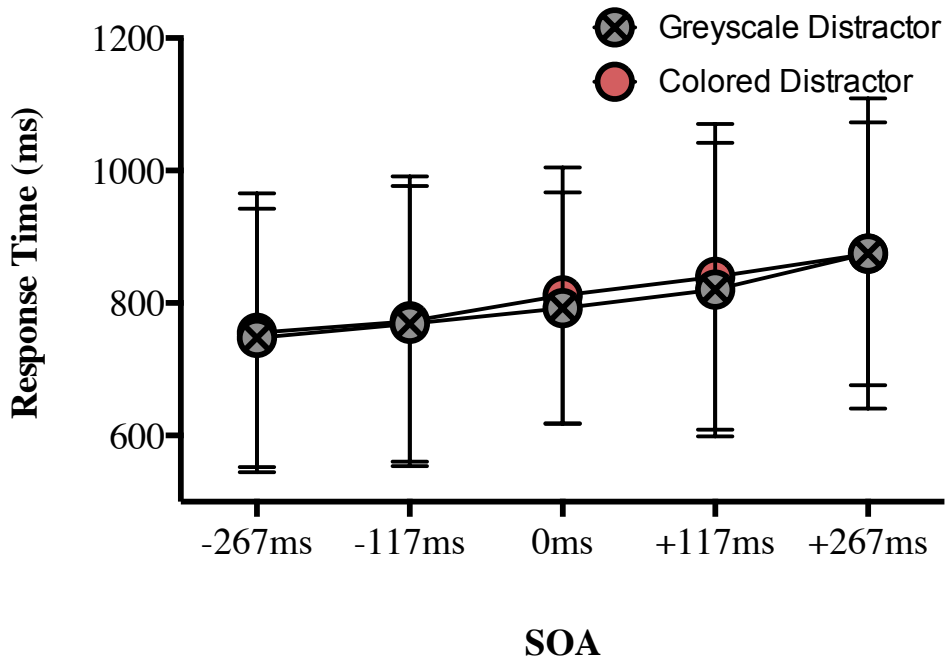

Supplement: S2 Fig — A two-way repeated measures ANOVA on Response Time with the factors of SOA (-267ms, -117ms, 0ms, +117ms, +267ms) and distractor type (greyscale, colored) showed a significant main effect of SOA (F(4, 72) = 37.437, p < 0.001), no effect of distractor type (F(1, 18) = 3.253, p = 0.088), and no interaction (F(4, 72) = 0.839, p = 0.505). (PDF) [file pone.0219725.s002.pdf]

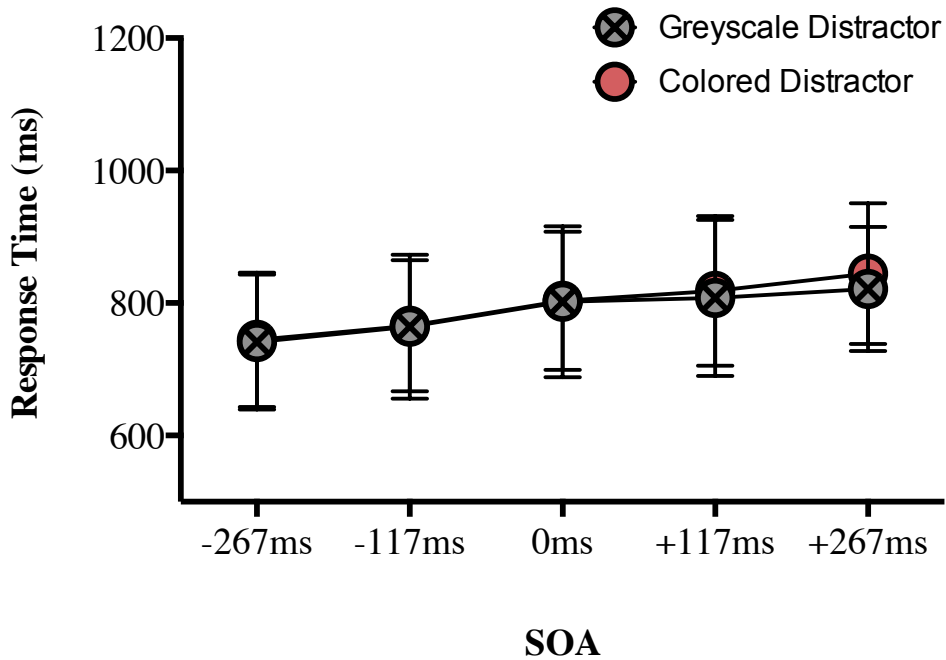

Supplement: S3 Fig — A two-way repeated measures ANOVA on Response Time with the factors of SOA (-267ms, -117ms, 0ms, +117ms, +267ms) and distractor type (greyscale, colored) showed a significant main effect of SOA (F(4, 72) = 36.272, p < 0.001), a significant main effect of distractor type (F(1, 18) = 5.23, p = 0.034), and no interaction (F(4, 72) = 1.133, p = 0.348). (PDF) [file pone.0219725.s003.pdf]
