# Supplementary material for: Late disruption of central visual field disrupts peripheral perception of form and color
Source: PLoS One. 2020 Jan 30;15(1):e0219725. doi: 10.1371/journal.pone.0219725 (PMC6991998; doi:10.1371/journal.pone.0219725)
Supplement: S1 Table — The Hit Rate was defined as the proportion of correct “same” responses on “same” trials, and the False Alarm Rate was defined as the proportion of “same” responses on “different” trials. Trials where eye-tracked participants failed to maintain fixation were not included in the final analysis. (PDF) [file pone.0219725.s004.pdf]

S1 Table. Hit and false alarm proportions in Experiment 1: Discriminating f

Greyscale Distractor					
SOA	Total Different Trials	Total Same Trials	Hit Rate	False Alarm Rate	*d'*
-267ms	725	710	0.78	0.31	1.29
-117ms	713	720	0.77	0.32	1.19
0ms	710	718	0.72	0.34	0.98
+117ms	718	713	0.65	0.44	0.56
+267ms	720	719	0.74	0.33	1.09
Colored Distractor					
SOA	Total Different Trials	Total Same Trials	Hit Rate	False Alarm Rate	*d'*
-267ms	715	713	0.80	0.33	1.29
-117ms	704	706	0.76	0.33	1.15
0ms	714	711	0.72	0.33	1.02
+117ms	714	717	0.65	0.41	0.61
+267ms	708	706	0.72	0.32	1.04
