# Supplementary material for: Late disruption of central visual field disrupts peripheral perception of form and color
Source: PLoS One. 2020 Jan 30;15(1):e0219725. doi: 10.1371/journal.pone.0219725 (PMC6991998; doi:10.1371/journal.pone.0219725)
Supplement: S2 Table — Asterisks indicate significance after Bonferroni correction for multiple comparisons (α = 0.05/10 = 0.005). (PDF) [file pone.0219725.s005.pdf]

S2 Table. Experiment 1: Discriminating form analysis.

			Uncorrected Comparisons (*p*)			
SOA	Mean	SEM	-117ms SOA	0ms SOA	+117ms SOA	+267ms SOA
-267ms	1.355	0.124	0.071	< 0.001*	< 0.001*	0.027
-117ms	1.218	0.116		0.016	< 0.001*	0.298
0ms	1.077	0.116			< 0.001*	0.560
+117ms	0.641	0.102				< 0.001*
+267ms	1.128	0.095				
