# Supplementary material for: Late disruption of central visual field disrupts peripheral perception of form and color
Source: PLoS One. 2020 Jan 30;15(1):e0219725. doi: 10.1371/journal.pone.0219725 (PMC6991998; doi:10.1371/journal.pone.0219725)
Supplement: S3 Table — The Hit Rate was defined as the proportion of correct “same” responses on “same” trials, and the False Alarm Rate was defined as the proportion of “same” responses on “different” trials. Trials where eye-tracked participants failed to maintain fixation were not included in the final analysis. (PDF) [file pone.0219725.s006.pdf]

S3 Table. Hit and false alarm proportions in Experiment 2: Discriminating color.

Greyscale Distractor					
SOA	Total Different Trials	Total Same Trials	Hit Rate	False Alarm Rate	*d'*
-267ms	734	738	0.68	0.33	0.91
-117ms	736	738	0.65	0.35	0.77
0ms	729	738	0.70	0.42	0.72
+117ms	739	731	0.63	0.37	0.65
+267ms	730	738	0.68	0.36	0.83
Colored Distractor					
SOA	Total Different Trials	Total Same Trials	Hit Rate	False Alarm Rate	*d'*
-267ms	734	734	0.74	0.38	0.95
-117ms	738	734	0.65	0.30	0.90
0ms	731	731	0.68	0.39	0.72
+117ms	737	735	0.63	0.43	0.52
+267ms	731	739	0.69	0.32	0.96
