# Supplementary material for: Late disruption of central visual field disrupts peripheral perception of form and color
Source: PLoS One. 2020 Jan 30;15(1):e0219725. doi: 10.1371/journal.pone.0219725 (PMC6991998; doi:10.1371/journal.pone.0219725)
Supplement: S4 Table — Asterisks indicate significance after Bonferroni correction for multiple comparisons (α = 0.05/10 = 0.005). (PDF) [file pone.0219725.s007.pdf]

S4 Table. Experiment 2: Discriminating color analysis.

			Uncorrected Comparisons (*p*)			
SOA	Mean	SEM	-117ms SOA	0ms SOA	+117ms SOA	+267ms SOA
-267ms	1.028	0.124	0.264	0.021	0.001*	0.419
-117ms	0.944	0.116		0.063	0.003*	0.686
0ms	0.814	0.116			0.094	0.047
+117ms	0.661	0.102				< 0.001*
+267ms	0.973	0.161				
