# Supplementary material for: Late disruption of central visual field disrupts peripheral perception of form and color
Source: PLoS One. 2020 Jan 30;15(1):e0219725. doi: 10.1371/journal.pone.0219725 (PMC6991998; doi:10.1371/journal.pone.0219725)
Supplement: S5 Table — The Hit Rate was defined as the proportion of correct “same” responses on “same” trials, and the False Alarm Rate was defined as the proportion of “same” responses on “different” trials. Trials where eye-tracked participants failed to maintain fixation were not included in the final analysis. (PDF) [file pone.0219725.s008.pdf]

**S5 Table. Hit and false alarm proportions in Experiment 3: Color discrimination with sin**

| Greyscale Distractor | | | | | |
|---|---|---|---|---|---|
| SOA | Total Different Trials | Total Same Trials | Hit Rate | False Alarm Rate | *d'* |
| -267ms | 756 | 755 | 0.75 | 0.30 | 1.21 |
| -117ms | 754 | 758 | 0.75 | 0.29 | 1.21 |
| 0ms | 759 | 759 | 0.74 | 0.41 | 0.88 |
| +117ms | 755 | 759 | 0.69 | 0.32 | 0.99 |
| +267ms | 762 | 758 | 0.80 | 0.35 | 1.22 |
| Colored Distractor | | | | | |
| SOA | Total Different Trials | Total Same Trials | Hit Rate | False Alarm Rate | *d'* |
| -267ms | 751 | 757 | 0.75 | 0.34 | 1.10 |
| -117ms | 759 | 762 | 0.77 | 0.35 | 1.12 |
| 0ms | 751 | 761 | 0.81 | 0.36 | 1.22 |
| +117ms | 755 | 768 | 0.71 | 0.37 | 0.87 |
| +267ms | 755 | 753 | 0.78 | 0.34 | 1.16 |

**nple shapes.**
