# Supplementary material for: Late disruption of central visual field disrupts peripheral perception of form and color
Source: PLoS One. 2020 Jan 30;15(1):e0219725. doi: 10.1371/journal.pone.0219725 (PMC6991998; doi:10.1371/journal.pone.0219725)
Supplement: S6 Table — Asterisks indicate significance after Bonferroni correction for multiple comparisons (α = 0.05/20 = 0.0025). (PDF) [file pone.0219725.s009.pdf]

S6 Table. Experiment 3: Color discrimination with simple shapes analysis A

			Uncorrected Comparisons (*p*)			
Greyscale Distractor						
SOA	Mean	SEM	-117ms SOA	0ms SOA	+117ms SOA	+267ms SOA
-267ms	1.295	0.123	0.990	0.001*	0.019	0.862
-117ms	1.297	0.114		0.007	0.088	0.821
0ms	0.952	0.126			0.171	0.004
+117ms	1.073	0.136				0.019
+267ms	1.314	0.130				

			Uncorrected Comparisons (*p*)			
Colored Distractor						
SOA	Mean	SEM	-117ms SOA	0ms SOA	+117ms SOA	+267ms SOA
-267ms	1.185	0.128	0.954	0.208	0.058	0.738
-117ms	1.192	0.113		0.274	0.013	0.739
0ms	1.330	0.123			0.002*	0.357
+117ms	0.931	0.109				0.003
+267ms	1.226	0.110				
