# Supplementary material for: Late disruption of central visual field disrupts peripheral perception of form and color
Source: PLoS One. 2020 Jan 30;15(1):e0219725. doi: 10.1371/journal.pone.0219725 (PMC6991998; doi:10.1371/journal.pone.0219725)
Supplement: S7 Table — Asterisks indicate significance after Bonferroni correction for multiple comparisons (α = 0.05/10 = 0.005). (PDF) [file pone.0219725.s010.pdf]

**S7 Table. Experiment 3: Color discrimination with simple shapes**					
Uncorrected Comparisons (*p*)					
SOA (greyscale)	-267ms SOA (colored)	-117ms SOA (colored)	0ms SOA (colored)	+117ms SOA (colored)	+267ms SOA (colored)
-267ms	0.275				
-117ms		0.382			
0ms			0.004*		
+117ms				0.182	
+267ms					0.326
